# Ginkgolide B inhibits platelet and monocyte adhesion in TNFα-treated HUVECs under laminar shear stress

**DOI:** 10.1186/s12906-018-2284-8

**Published:** 2018-07-20

**Authors:** Ming Zhang, Jie Sun, Beidong Chen, Yanyang Zhao, Huan Gong, Yun You, Ruomei Qi

**Affiliations:** 1 MOH Key Laboratory of Geriatrics, Beijing Hospital, National Center of Gerontology, Beijing, China; 2 Graduate School of Peking Union Medical College, Chinese Academy of Medical Sciences, Beijing, China; 3 Institute of Chinese Materia Medica, China Academy of Chinese Medical Sciences, Beijing, China

**Keywords:** Ginkgolide B, Endothelial cells, Platelets, VCAM-1, VE-cadherin, Shear stress

## Abstract

**Background:**

Endothelial cells are sensitive to changes in both blood components and mechanical stimuli. Endothelial cells may undergo phenotypic changes, such as changes in adhesion protein expression, under different shear stress conditions. Such changes may impact platelet and monocyte adhesion to endothelial cells. This phenomenon is linked to chronic vascular inflammation and the development of atherosclerosis. In the present study, we investigated the effects of ginkgolide B on platelet and monocyte adhesion to human umbilical vein endothelial cells (HUVECs) under different conditions of laminar shear stress.

**Methods:**

Platelet and monocyte adhesion to endothelial cells was determined by the Bioflux 1000. HUVECs were incubated with ginkgolide B or aspirin for 12 h, and then TNFα was added for 2 h to induce the inflammatory response under conditions of 1 and 9 dyn/cm^2^ laminar shear stress. The protein expression was analyzed by Western blot.

**Results:**

The number of platelets that adhered was greater under conditions of 1 dyn/cm^2^ than under conditions of 9 dyn/cm^2^ of laminar shear stress (74.8 ± 19.2 and 59.5 ± 15.1, respectively). Ginkgolide B reduced the tumor necrosis factor α (TNFα)-induced increase in platelet and monocyte adhesion to HUVECs at 1 and 9 dyn/cm^2^ of laminar shear stress. In TNFα-treated HUVECs, the number of monocytes that adhered was greater under conditions of 1 dyn/cm^2^ of laminar shear stress compared with 9 dyn/cm^2^ (29.1 ± 4.9 and 22.7 ± 3.7, respectively). Ginkgolide B inhibited the TNFα-induced expression of vascular cell adhesion molecule-1(VCAM-1), VE-cadherin, and Cx43 in HUVECs at 1 and 9 dyn/cm^2^. The expression of these proteins was not different between 1 and 9 dyn/cm^2^.

**Conclusions:**

Ginkgolide B suppressed platelet and monocyte adhesion under different conditions of laminar shear stress. Moreover, ginkgolide B reduced VCAM-1, VE-cadherin and Cx43 expression in TNFα-treated HUVECs under laminar shear stress. This suggested that ginkgolide B might shed light on the treatment of inflammation in atherosclerosis.

## Background

Endothelial cells are the initial barriers on the vessel wall. Endothelial cell dysfunction is a primary cause of cardiovascular disease. Endothelial cells are sensitive to changes in both blood components and mechanical stimuli [1–3]. In humans, normal physiological flow ranges from 10 to 50 dyn/cm^2^ and is highly pulsatile in arteries and ~ 10-fold less with minimal pulsation in veins [4]. High shear stress that results from laminar flow promotes endothelial cell survival and quiescence, alignment in the direction of the flow, and the secretion of substances that promote vasodilation and anticoagulation [5, 6]. Low shear stress or changes in the direction of shear stress, as found in turbulent flow, promotes vasoconstriction, coagulation, and platelet aggregation [7, 8]. The precise mechanisms by which endothelial cells sense shear stress to affect cell-cell interactions are still not completely understood. Endothelial cells may undergo phenotypic changes, such as changes in adhesion protein expression, under different shear stress conditions. Such changes may impact platelet and monocyte adhesion to endothelial cells. This phenomenon is linked to chronic vascular inflammation and the development of atherosclerosis [9–11].

In the physiological state, endothelial cells maintain vessel integrity through junction proteins. VE-cadherin is a calcium-dependent cell-cell adhesion protein that is composed of five extracellular cadherin repeats and a transmembrane region [12]. VE-cadherin antibodies were shown to increase monolayer permeability in cultured cells [13]. We recently reported that VE-cadherin was involved in monocyte translocation in oxidized low-density lipoprotein (ox-LDL)-treated endothelial cells. Treatment with VE-cadherin siRNA reduced monocyte translocation in ox-LDL-treated endothelial cells [14]. Cx43 is also a junction protein that belongs to the gap junction protein family. Gap junctions play a role in intercellular communication between cells to regulate cell death, proliferation, and differentiation [15]. Gap junctions are involved in connecting adjacent cells to permit the exchange of low-molecular-weight molecules, such as ions and secondary messengers, to maintain homeostasis [16, 17].

Tumor necrosis factor α (TNFα) is a member of the cytokine family and involved in various inflammatory processes. TNFα is implicated in several human diseases, including atherosclerosis and cardiovascular disease. It is a potent inducer of nuclear factor κB (NF-кB) signaling, which is involved in the transcription of many inflammatory proteins. TNFα is also involved in endothelial cell injury under pathological conditions, such as atherosclerosis [18].

Ginkgolide B is a *Ginkgo biloba* leaf extract that can completely bind platelet-activating factor receptor (PAFR) and inhibit platelet activation [19]. Our recent studies showed that ginkgolide B inhibited inflammatory protein expression that was induced by ox-LDL in human umbilical vein endothelial cells (HUVECs) [20, 21]. However, remaining unknown is whether ginkgolide B inhibits platelet and monocyte adhesion to endothelial cells under different shear stress conditions. In the present study, we investigated the effects of ginkgolide B on platelet and monocytes adhesion to endothelial cells and phenotypic changes under different laminar shear stress conditions.

## Methods

### Materials

Ginkgolide B (95% purity) was purchased from Daguanyuan Company (Xuzhou, Jiangsu, China). Mouse tail type I collagen were purchased from Sigma-Aldrich (St. Louis, MO, USA). Monoclonal anti-connexin 43 antibody, polyclonal anti-VCAM-1 antibody and monoclonal anti-actin antibody were purchased from Santa Cruz Biotechnology (Santa Cruz, CA, USA). Monoclonal anti-VE-cadherin antibody was purchased from Abcam (Boston, MA, USA). Bioflux 48-well plates (1–20 dyn/cm^− 2^; 910–0047) were purchased from Fluxion Biosciences (South San Francisco, CA, USA).

### Preparation of platelets

Fresh citrate anti-coagulated venous blood was obtained from human donors who had not taken any medication for a minimum of 2 weeks before blood collection. The blood was centrifuged at 400 × g for 15 min to obtain platelet-rich plasma (PRP). The PRP was washed twice in Tyrode’s/HEPES buffer with 2 mM ethylene glycol tetraacetic acid (EGTA). Platelets were suspended in Tyrode’s/HEPES buffer at a concentration of 2 × 108 cells/ml [22].

### Preparation of monocytes

The THP-1 human monocytic cell line was obtained from the American Type Culture Collection (Manassas, VA, USA). The cells were maintained at 37 °C in RPMI 1640 medium supplemented with 10% fetal calf serum (FCS), 100 IU penicillin, 100 μg/ml streptomycin, and 2 mM L-glutamine in a humidified 5% carbon dioxide atmosphere [23].

### Preparation and culture of human umbilical vein endothelial cells

HUVECs were purchased from ScienCell Research Laboratories (Carlsbad, CA, USA). HUVECs were redissolved in a 37 °C constant temperature water bath. The cells were cultured in M199 medium that contained 10% fetal bovine serum (Gibco, NY, USA), 2 mM glutamine, 100 U/ml penicillin, 100 μg/ml streptomycin, and 20 ng/ml endothelial growth factor (R&D, Minneapolis, MN, USA) in an incubator at 37 °C and 5% CO_2_. Cells up to passage 4 were used in the experiments [24].

### Platelet and monocyte adhesion to HUVECs assay

The Bioflux 1000 system (Fluxion Biosciences Inc., CA, USA) was used in the present study. The flow experiments were performed as previously described [25]. Briefly, the microfluidic channel was coated with type I collagen (20 μg/ml) that was dissolved in 0.02 M acetic acid. HUVECs (3 × 10^7^) were seeded in microfluidic well plates until > 90% of the cells covered the well plate that was used for the experiments. HUVECs were treated with ginkgolide B (0.6 mg/ml) or aspirin (1 mM) for 12 h, and then TNFα was added for another 2 h under conditions of 1 and 9 dyn/cm^2^ laminar shear stress. The platelet suspension (300 μl, 2 × 10^8^) or monocyte suspension (300 μl, 5 × 10^6^) was added to the input channel for 45 min for cell adhesion under condition of 0.2 dyn/cm^2^. Images were captured by a Nikon Ti100 CCD camera in five locations in the flow chamber and analyzed using Bioflux Montage software.

### Western blot

HUVECs were incubated with ginkgolide B (0.6 mg/ml) or aspirin (1 mM) for 12 h, and then TNFα (20 ng/ml) was added for 2 h under conditions of 1 and 9 dyn/cm^2^ laminar shear stress. The platelet or monocyte suspension was perfused for 45 min under condition of 0.2 dyn/cm^2^ for adhesion. After that the microfluidic channel was washed with PBS to remove unadhered platelets or monocytes for 5 min under condition of 2 dyn/cm^2^, and then lysis buffer (1% Triton X-100, 100 mM Tris/HCl [pH 7.2], 50 mM NaCl, 5 mM ethylenediaminetetraacetic acid [EDTA], 5 mM EGTA, 1 μM phenylmethylsulfonyl fluoride [PMSF], and 100 μg/ml leupeptin) was added. Lysates were centrifuged at 12000 × *g* at 4 °C for 5 min. To obtain sufficient protein, three parallel microfluidic flow channels were used in each group. The cell lysates were separated by 10% sodium dodecyl sulfatepolyacrylamide gel electrophoresis (SDS-PAGE), and transferred to a polyvinylidene difluoride membrane (Millipore, Billerica, MA, USA. Primary antibody incubations were performed overnight at 4 °C. Horseradish peroxidase-conjugated secondary antibody was applied for 1 h at room temperature and developed using Super Signal developing reagent (Pierce, Thermo Scientific). Blot densitometry was then performed, and the bands were analyzed using the Gene Genius Bio Imaging System.

### Statistical analysis

Quantitative data are presented as mean ± SEM. Significant differences between two groups were analyzed by two-tail unpaired Student’s *t*-test. All of the calculations were performed using SPSS 18.0 software (Armonk, NY, USA). Values of *p* < 0.05 were considered statistically significant.

## Results

### Ginkgolide B decreases platelet adhesion to TNFα-treated HUVECs under conditions of laminar shear stress

**Fig. 1:**
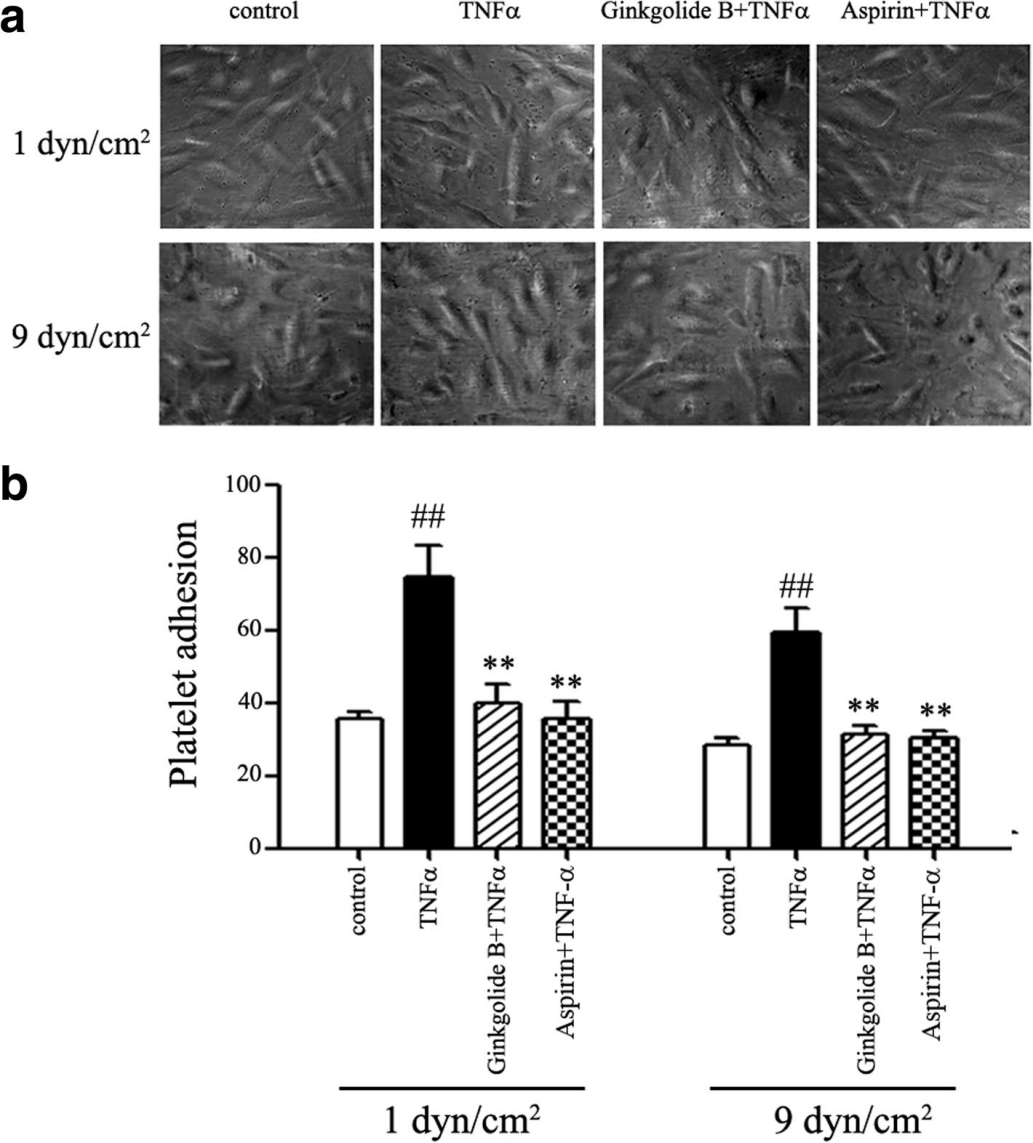
Ginkgolide B reduced platelet adhesion in TNFα-treated HUVECs under different conditions of laminar shear stress. HUVECs were incubated with ginkgolide B (0.6 mg/ml) or aspirin (1 mM) for 12 h, and then TNFα (20 ng/ml) was added for another 2 h. The platelet suspension was then added to the input well under conditions of 1 and 9 dyn/cm^2^ of laminar shear stress for 45 min. The data were obtained from three independent experiments. **a** Ginkgolide B decreased the number of platelets that adhered to TNFα-treated HUVECs at 1 and 9 dyn/cm^2^. **b** Platelet adhesion to HUVECs. ^##^*p* < 0.01, significant difference between TNFα-treated and -untreated HUVECs. **p < 0.01, significant difference between TNFα-treated cells and ginkgolide B- and aspirin-treated cells

To investigate the interaction between platelets and HUVECs, platelet adhesion was determined in TNFα-treated HUVECs under conditions of 1 and 9 dyn/cm^2^ of shear stress. Our previous studies showed that 0.6 mg/ml ginkgolide B and 1 mM aspirin significantly inhibited the inflammatory response of endothelial cells. Therefore, we applied the same doses of ginkgolide B and aspirin in the present study. As shown in Fig. 1, at 1 dyn/cm^2^, the number of platelets that adhered was 74.8 ± 19.2 and 36.1 ± 4.4 in TNFα-treated and -untreated HUVECs, respectively. The number of platelets that adhered was 39.5 ± 12.3 in ginkgolide B-treated HUVECs. We used aspirin (1 mM) as a control. Aspirin treatment also reduced the number of platelets that adhered (35.8 ± 10.1) in TNFα-treated HUVECs. At 9 dyn/cm^2^, the number of platelets that adhered was 59.5 ± 15.1 and 28.8 ± 3.7 in TNFα-treated and -untreated HUVECs. The number of platelets that adhered was 31.5 ± 4.9 and 32.1 ± 4.3 in the ginkgolide B- and aspirin-treated groups, respectively.

### Ginkgolide B suppresses VCAM-1, VE-cadherin, and Cx43 expression in TNFα-treated HUVECs that present platelet adhesion under conditions of laminar shear stress

**Fig. 2:**
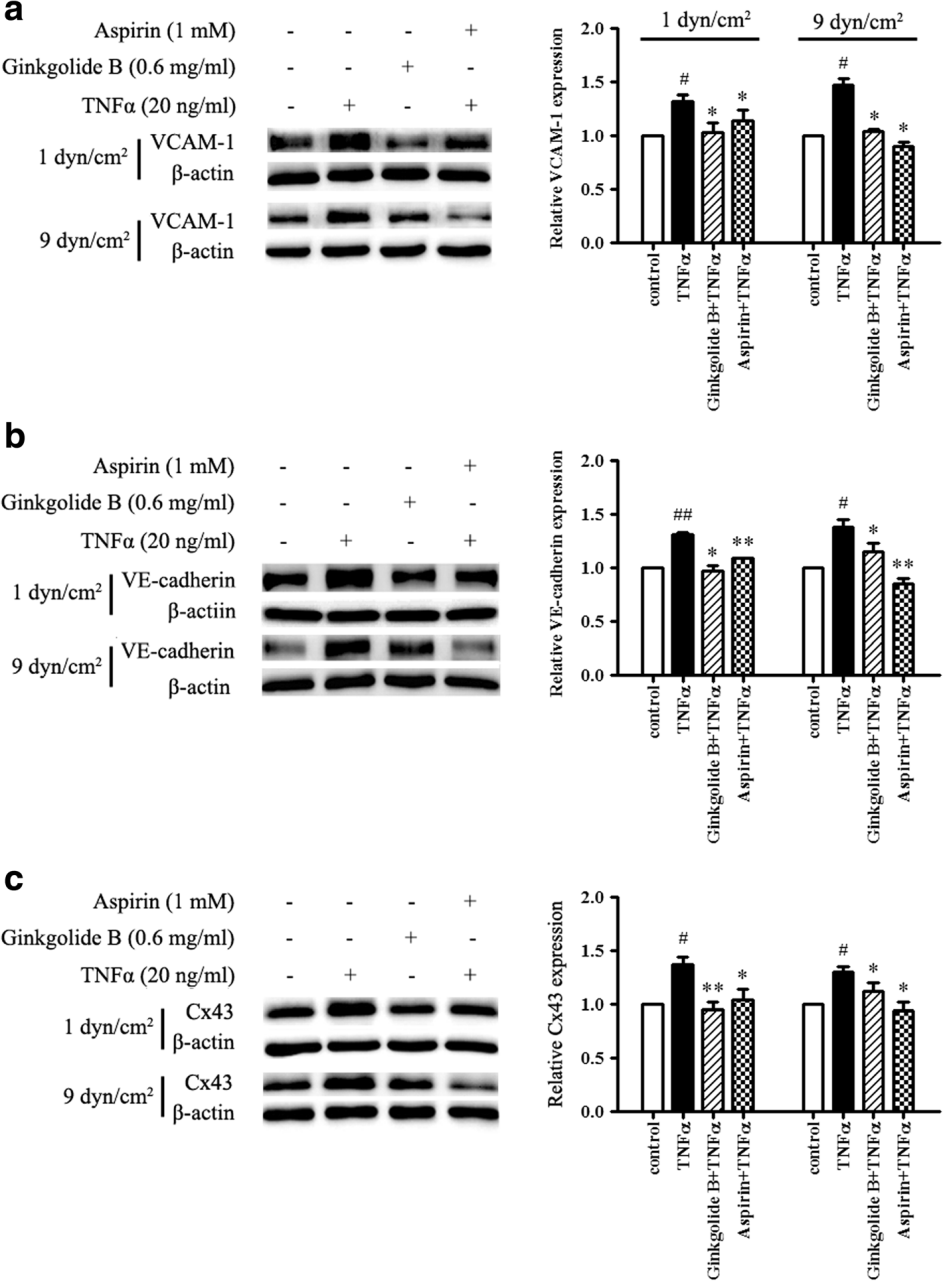
Ginkgolide B suppressed VCAM-1, VE-cadherin, and Cx43 expression in TNFα-treated HUVECs that presented platelet adhesion under different conditions of laminar shear stress. HUVECs were incubated with ginkgolide B (0.6 mg/ml) or aspirin (1 mM) for 12 h, and then TNFα (20 ng/ml) was added for another 2 h. The platelet suspension was then added to the input well at 1 and 9 dyn/cm^2^ of laminar shear stress for 45 min. Unadhered platelets were removed by the addition of PBS buffer for 2 min. Lysates were then collected by the addition of lysis buffer. Protein expression was analyzed by Western blot. The data were obtained from three independent experiments. **a** Ginkgolide B inhibited TNFα-induced VCAM-1 expression at 1 and 9 dyn/cm^2^. **b** Ginkgolide B decreased TNFα-induced VE-cadherin expression at 1 and 9 dyn/cm^2^. **c** Ginkgolide B reduced TNFα-induced Cx43 expression at 1 and 9 dyn/cm^2^. ^#^*p* < 0.05, significant difference between TNFα-treated and -untreated HUVECs. **p < 0.01, significant difference between TNFα-treated cells and ginkgolide B- and aspirin-treated cells

We first investigated the effects of ginkgolide B on VCAM-1 expression under laminar shear stress. As shown in Fig. 2a, at 1 dyn/cm^2^, VCAM-1 expression increased by 31.7% ± 2.9% in TNFα-treated HUVECs compared with the control. Ginkgolide B and aspirin completely inhibited TNFα-induced VCAM-1 expression. VCAM-1 expression increased by 46.9% ± 10.1% in TNFα-treated HUVECs under conditions of 9 dyn/cm^2^ of laminar shear stress. Ginkgolide B (0.6 mg/ml) almost completely attenuated TNFα-induced VCAM-1 expression. Similar results were found in the aspirin-treated group.

We next evaluated the effects of ginkgolide B on the expression of the tight junction proteins VE-cadherin and Cx43 under different conditions of laminar shear stress. As shown in Fig. 2b and c, TNFα treatment increased VE-cadherin expression by 31.8% ± 2.2% and 38.9 ± 9.1% under conditions of 1 and 9 dyn/cm^2^ of laminar shear stress, respectively. Ginkgolide B (0.6 mg/ml) and aspirin (1 mM) completely abolished TNFα-induced VE-cadherin expression at 1 and 9 dyn/cm^2^. Cx43 expression increased by 37.7% ± 9.8 and 30.5% ± 6.1% in TNFα-treated HUVECs that presented platelet adhesion at 1 and 9 dyn/cm^2^, respectively. Ginkgolide B (0.6 mg/ml) and aspirin (1 mM) significantly inhibited TNFα-induced Cx43 expression at both 1 and 9 dyn/cm^2^.

### Ginkgolide B decreases monocyte adhesion to TNFα-treated HUVECs under conditions of laminar shear stress

**Fig. 3:**
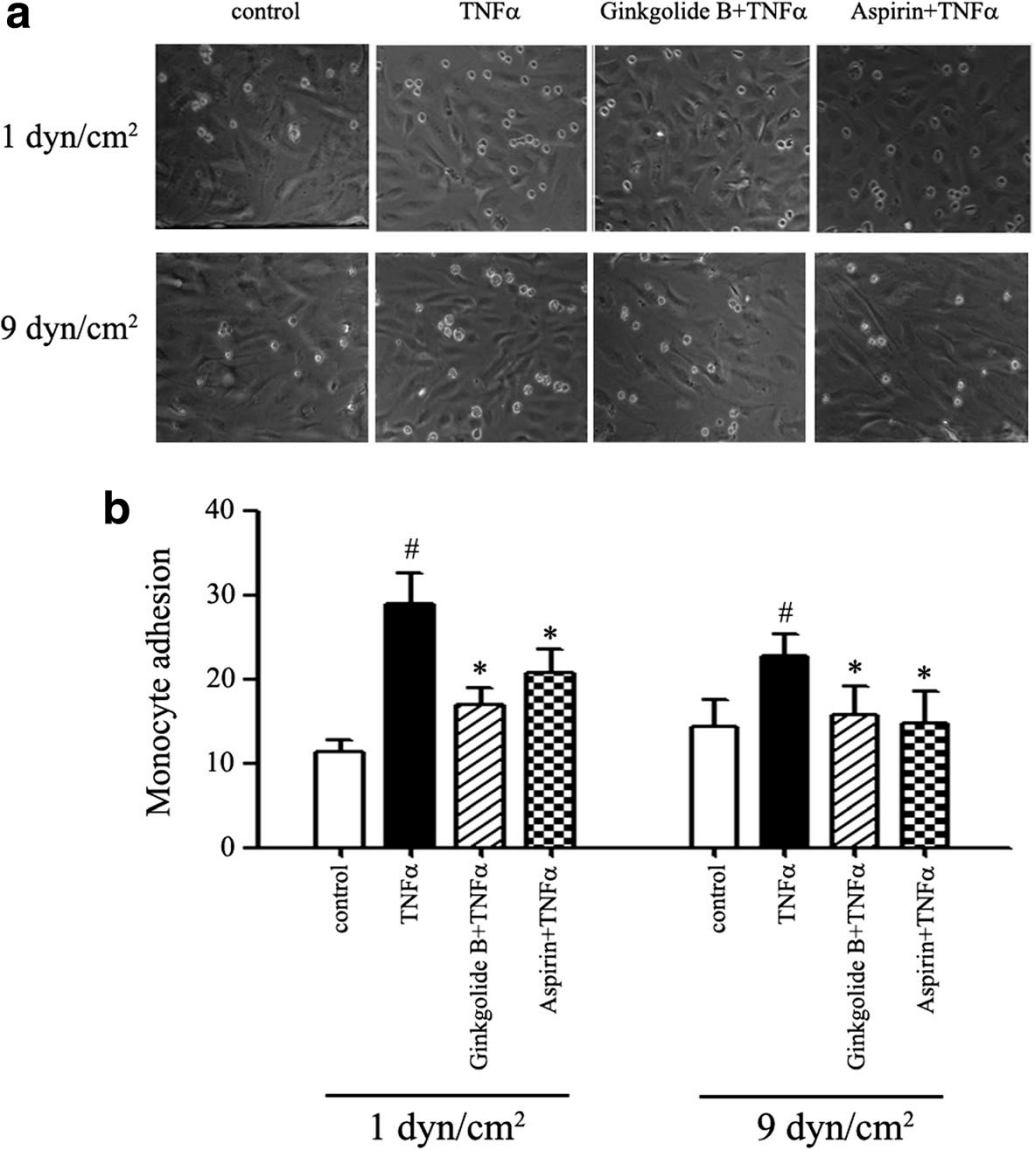
Ginkgolide B reduced monocyte adhesion to TNFα-treated HUVECs under different conditions of laminar shear stress. HUVECs were incubated with ginkgolide B (0.6 mg/ml) or aspirin (1 mM) for 12 h, and then TNFα (20 ng/ml) was added for another 2 h. The monocyte suspension was then added to the input well at 1 and 9 dyn/cm^2^ of laminar shear stress for 45 min. The data were obtained from three independent experiments. **a** Ginkgolide B reduced TNFα-induced monocyte adhesion to HUVECs at 1 and 9 dyn/cm^2^. **b** Monocyte adhesion to HUVECs. ^#^*p* < 0.05, significant difference between TNFα-treated and -untreated HUVECs. *p < 0.01, significant difference between TNFα-treated cells and ginkgolide B- and aspirin-treated cells

Furthermore, monocyte adhesion to endothelial cells was evaluated under conditions of laminar shear stress. As shown in Fig. 3a, at 1 dyn/cm^2^, the number of monocytes that adhered was 29.1 ± 4.9 in TNFα-treated HUVECs. The number of monocytes that adhered was 11.3 ± 1.9 in the control group. At 9 dyn/cm^2^, the number of monocytes that adhered was 22.7 ± 3.7 in TNFα-treated HUVECs. The number of monocytes that adhered was 14.3 ± 4.5 in the control group. Ginkgolide B (0.6 mg/ml) inhibited monocyte adhesion to TNFα-treated HUVECs. The number of monocytes that adhered was 17.0 ± 2.8 at 1 dyn/cm^2^ and 15.7 ± 4.8 at 9 dyn/cm^2^. In the aspirin-treated group, the number of monocytes that adhered was 20.7 ± 1.8 and 14.7 ± 5.4 at 1 and 9 dyn/cm^2^, respectively. Significant differences were found between TNFα-treated cells and ginkgolide B- and aspirin-treated cells.

### Ginkgolide B suppresses VCAM-1, VE-cadherin, and Cx43 expression in TNFα-treated HUVECs that present monocyte adhesion under conditions of laminar shear stress

**Fig. 4:**
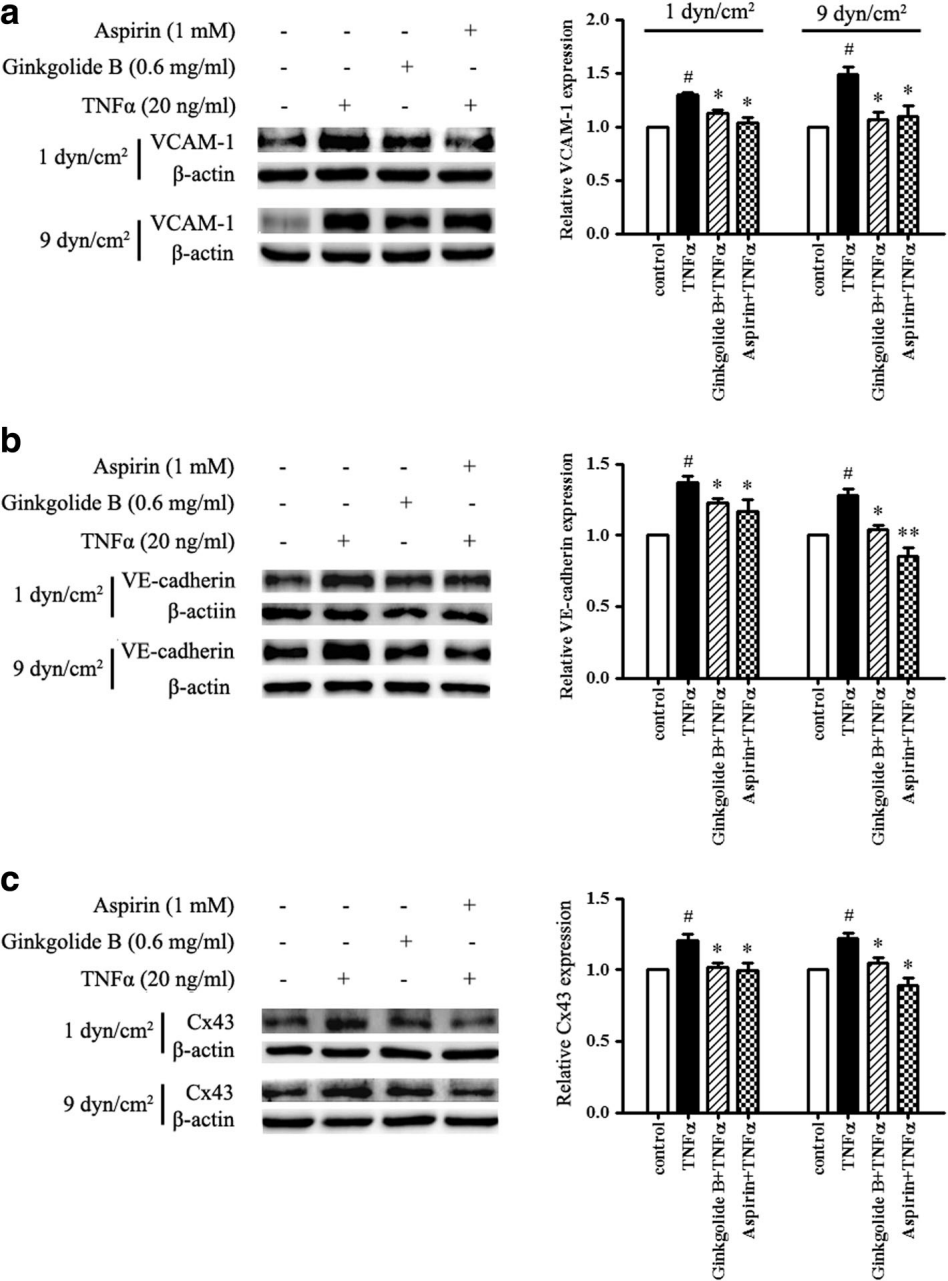
Ginkgolide B suppressed VCAM-1, VE-cadherin, and Cx43 expression in TNFα-treated HUVECs that presented monocyte adhesion under different conditions of laminar shear stress. HUVECs were incubated with ginkgolide B (0.6 mg/ml) or aspirin (1 mM) for 12 h, and then TNFα (20 ng/ml) was added for another 2 h. The monocyte suspension was then added to the input well at 1 and 9 dyn/cm^2^ of laminar shear stress for 45 min. Unadhered monocytes were removed by the addition of PBS buffer for 2 min. Lysates were then collected by the addition of lysis buffer. Protein expression was analyzed by Western blot. **a** Ginkgolide B inhibited TNFα-induced VCAM-1 expression at 1 and 9 dyn/cm^2^. **b** Ginkgolide B decreased TNFα-induced VE-cadherin expression at 1 and 9 dyn/cm^2^. **c** Ginkgolide B reduced TNFα-induced Cx43 expression at 1 and 9 dyn/cm^2^. ^#^*p* < 0.05, significant difference between TNFα-treated and -untreated HUVECs. *p < 0.05, **p < 0.01, significant difference between TNFα-treated cells and ginkgolide B- and aspirin-treated cells

We also evaluated the effects of ginkgolide B on adhesion proteins and tight junction proteins under conditions of laminar shear stress and monocyte adhesion in HUVECs. As shown in Fig. 4a-c, VCAM-1 expression increased by 29.8% ± 2.9% at 1 dyn/cm^2^ and by 48.6% ± 10.1% at 9 dyn/cm^2^ in TNFα-treated HUVECs. Ginkgolide B inhibited TNFα-induced VCAM-1 expression at both 1 and 9 dyn/cm^2^. Similar results were found in aspirin-treated HUVECs.

TNFα treatment increased VE-cadherin expression by 36.9% ± 6.6 and 28.2% ± 6.0% at 1 and 9 dyn/cm^2^, respectively. Ginkgolide B and aspirin reduced TNFα-induced VE-cadherin expression at 1 and 9 dyn/cm^2^. Cx43 expression increased by 20.9% ± 7.1 and 21.8% ± 5.4% at 1 and 9 dyn/cm^2^ in TNFα-treated HUVECs, respectively. Both ginkgolide B and aspirin completely suppressed TNFα-induced Cx43 expression at 1 and 9 dyn/cm^2^.

## Discussion

Platelets, monocytes, and endothelial cells interact in pathological states. Platelets release several inflammatory mediators, such as platelet factor 4 (PF4), regulated on activation, normal T-cell expressed and secreted chemokine (RANTES), P-selectin, and CD40 ligand, to recruit monocytes to the site of damaged endothelial cells [26–28]. The adhesion of platelets through the expression of adhesion proteins can also elicit an inflammatory response in endothelial cells [29]. Monocyte adhesion to endothelial cells and enter intima where phagocytosis lipids and transform into macrophage and foam cells [30–32]. This process is involved in plaque formation in atherosclerosis. Therefore, the inhibition of platelet and monocyte adhesion to endothelial cells might be a strategy for preventing atherosclerosis.

In the present study, we used the Bioflux 1000 microfluidic device to investigate platelet and monocyte adhesion to endothelial cells under different conditions of laminar shear stress. High shear stress in the laminar flow promotes endothelial cell survival and anticoagulation. Low shear stress in the laminar flow promotes endothelial cell proliferation, apoptosis, and coagulation [33]. However, the precise mechanisms by which these processes occur are still unknown. To determine the effects of different conditions of shear stress on the interaction between platelets/monocytes and endothelial cells, two conditions of shear stress were used (1 and 9 dyn/cm^2^). Laminar shear stress at 1 dyn/cm^2^ reflects a pathological state, and 9 dyn/cm^2^ reflects a physiological state [34]. Areas of low shear stress in vessels may be linked to endothelial cell dysfunction, reflected by lower nitric oxide and prostacyclin production. The present results showed that the number of platelets that adhered to HUVECs at 1 dyn/cm^2^ was greater than the number that adhered at 9 dyn/cm^2^. Monocyte adhesion presented a similar trend. The number of monocytes that adhered to HUVECs at 1 dyn/cm^2^ was greater than the number that adhered at 9 dyn/cm^2^. These results support the hypothesis that platelets and monocytes under conditions of low shear stress easily adhere to endothelial cells. We also observed phenotypic changes in endothelial cells under conditions of low and high shear stress. The expression of VCAM-1, VE-cadherin, and Cx43 was not different between 1 and 9 dyn/cm^2^. This implies that 9 dyn/cm^2^ of laminar shear stress does not impact TNFα-induced VCAM-1, VE-cadherin, or Cx43 expression. Both ginkgolide B and aspirin exerted protective actions against the expression of these proteins that was induced by TNFα under conditions of laminar shear stress. This is consistent with our previous study, in which increases in VE-cadherin and Cx43 expression were linked to monocyte migration that was induced by ox-LDL. Furthermore, the knockdown of VE-cadherin and Cx43 gene expression by siRNA decreased the number of monocytes that migrated in ox-LDL-treated HUVECs [14]. This suggests VE-cadherin and Cx43 mediate monocyte migration, and this phenomenon might occur independently of their function at cell junctions. In the present study, platelet and monocyte adhesion decreased under conditions of high shear stress, but the underlying mechanism needs clarification.

Growing evidence has shown the protective action of ginkgolide B on cardiovascular and nervous system diseases. Our previous studies showed that ginkgolide B inhibited platelet aggregation and reduced CD40L, RANTES and PF4 secretion induced by thrombin and collagen. Moreover, ginkgolide B treatment decreased platelet adhesion on aortic plaque in Apo E gene defective mice [20]. Recent a study reported that ginkgolide B promoted microglia/macrophage transferring from inflammatory M1 phenotype to anti-inflammatory phenotype M2 in vivo and in vitro [35]. In recent years *Ginkgo biloba* extracts have been widely used as a phytomedicine in Europe and in the United States. These studies demonstrated that ginkgolide B might be a promising drug in clinic application.

## Conclusion

In conclusion, we found that platelet and monocyte adhesion was stronger under conditions of 1 dyn/cm^2^ of laminar shear stress compared with 9 dyn/cm^2^. Ginkgolide B inhibited TNFα-induced platelet and monocyte adhesion to endothelial cells and attenuated VCAM-1, VE-cadherin, and Cx43 expression under conditions of laminar shear stress. No differences in the expression of these proteins were found between 1 and 9 dyn/cm^2^. These findings suggest that ginkgolide B might shed light on the treatment of inflammation in atherosclerosis.

## Abbreviations

NF-кB: Nuclear factor κB
TNFα: Tumor necrosis factor α
EDTA: Ethylenediaminetetraacetic acid
EGTA: Ethylene glycol tetraacetic acid
HUVECs: Human umbilical vein endothelial cells
ox-LDL: Oxidized low-density lipoprotein
PMSF: Phenylmethylsulfonyl fluoride
RANTES: Regulated on activation, normal T-cell expressed and secreted chemokine
VCAM-1: Vascular cell adhesion molecule-1
